# A target-driven visual navigation method based on intrinsic motivation exploration and space topological cognition

**DOI:** 10.1038/s41598-022-07264-7

**Published:** 2022-03-02

**Authors:** Xiaogang Ruan, Peng Li, Xiaoqing Zhu, Pengfei Liu

**Affiliations:** 1 Faculty of Information Technology, Beijing University of Technology, Beijing, China; 2 Beijing Key Laboratory of Computational Intelligence and Intelligent System, Beijing, China

**Keywords:** Mathematics and computing, Computer science

## Abstract

Target-driven visual navigation is essential for many applications in robotics, and it has gained increasing interest in recent years. In this work, inspired by animal cognitive mechanisms, we propose a novel navigation architecture that simultaneously learns exploration policy and encodes environmental structure. First, to learn exploration policy directly from raw visual input, we use deep reinforcement learning as the basic framework and allow agents to create rewards for themselves as learning signals. In our approach, the reward for the current observation is driven by curiosity and calculated by a count-based approach and temporal distance. While agents learn exploration policy, we use temporal distance to find waypoints in observation sequences and incrementally describe the structure of the environment in a way that integrates episodic memory. Finally, space topological cognition is integrated into the model as a path planning module and combined with a locomotion network to obtain a more generalized approach to navigation. We test our approach in the DMlab, a visually rich 3D environment, and validate its exploration efficiency and navigation performance through extensive experiments. The experimental results show that our approach can explore and encode the environment more efficiently and has better capability in dealing with stochastic objects. In navigation tasks, agents can use space topological cognition to effectively reach the target and guide detour behaviour when a path is unavailable, exhibiting good environmental adaptability.

## Introduction

Humans and many animals can learn a variety of skills by interacting with their environment, and one useful skill is navigation^1^. Navigation has been widely studied in psychology and neuroscience since 1948 when Tolman^2^ introduced the concept of cognitive maps to explain the detour behaviour of rats. Until now, there has been no clear answer to the exact form of cognitive map. However, it is undeniable that animals can build a cognitive model of their environment from raw sensory input and use it to support subsequent actions such as finding food, shelter, or a mate.

Recently, deep reinforcement learning (DRL)^3^, a method that integrates the perceptual capability of deep learning (DL)^4^ with the decision-making capability of reinforcement learning (RL)^5^, has been used to solve control tasks in high-dimensional state space. Relying on a learning framework that can create a direct mapping from raw sensory input to action output, DRL has impressive results in target-driven navigation tasks. Zhu^6^ combined pretrained ResNet with a Siamese actor-critic architecture to accomplish target-driven visual navigation. This approach has a good generalization to new targets, but it lacks memory units and performs poorly in complex environments. Mnih^7^ proposed an asynchronous RL method and used it to train a model that was combined with long short-term memory (LSTM)^8^ network, for navigating in 3D mazes. Jaderbery^9^ studied the effects of various auxiliary tasks and found that denser reward feedback was helpful in learning navigation policy. Mirowski^3^ constructed a stacked LSTM framework that learns target-driven behaviour in conjunction with depth prediction and loop closure classification tasks and showed that data efficiency and task performance can be greatly improved when additional navigation-related signals are provided. To better transfer learned navigation skills to new environments, Ye^10^ introduced a model that includes a custom object recognition module. In this way, the agent can recognize a target regardless of where the image of the object comes from. Although the agent can reach 4 different targets in a scene, it is still not applicable to new environments. Yang^11^ proposed a framework that combines a pretrained ResNet with a word encoder to solve the generalization problem in visual semantic navigation. In their setup, they used a graph convolutional network (GCN) to encode semantic priors. It is worth noting that this semantic approach is not a good choice for visual navigation tasks, since the target is specified by the image rather than the semantic label. Devo^12^ proposed a novel architecture consisting of two networks, the first of which aims to explore the environment and the other at locating the target. The experimental results show that the agent can transfer learned skills to new environments without fine-tuning. These methods have proven that DRL is an effective framework to learn navigation policy, only through vision. However, mapless methods require retraining or at least fine-tuning the model to deal with changes in the environment. In the real world, this operation can be time-consuming or even impossible to complete. Therefore, we want to encode the structure of the environment while the agent learns control policy to better deal with the problems posed by dynamic elements. To cover the state space as soon as possible, we prefer to obtain an exploration policy during the interaction.

As we mentioned above, target-driven behaviour can be obtained based on specified targets and corresponding rewards in the environment; however, what should drive the exploration policy? By reading the literature on cognitive mechanisms in animals^13–15^, we found that curiosity is one of the motivations for animals to spontaneously explore their environment. Many AI agents take curiosity as an internal mechanism to push them to make sense of the world. Bellemare^16^ proposed a pseudo-count method that generalizes count-based exploration to the non-tabular case, and this method improves the efficiency of agent exploration in some difficult games, especially the game named Montezuma’s Revenge. Ostrovski^17^ replaced the model providing the pseudo-count in Bellemare’s method with PixelCNN and showed excellent exploration performance in many Atari games. In addition, they found that the mixed Monte Carlo update is a powerful facilitator for exploration. Tang^18^ combined a hash table with classical count-based exploration to compute a novelty bonus for states, and this combination allowed the method to reach near state-of-the-art performance on various continuous DRL benchmarks. Houthooft^19^ introduced an exploration method that creates intrinsic reward by maximizing the information gain of an agent’s beliefs about the dynamics of the environment, which shows superiority in simple video games but still struggle in complex environments. Relying on the theory that new states are more easily distinguished from other states, Fu^20^ used an exemplar model to detect novelty during interaction and combined it with a count-based method to guide exploration in egocentric observation. Pathak^21^ proposed an intrinsic curiosity module (ICM) that calculates intrinsic rewards based on prediction errors to push the agent in VizDoom and Super Mar Bros to explore the environment more efficiently, but it does not work when the agent observes something unpredictable.

The ICM model has given us some insight in building a curiosity-driven approach to exploration, but since predicting the future is harder than retrieving things from memory, we do not use prediction errors as a source of reward. Our reward function is related to episode memory^22^ and consists of two parts: (1) the frequency with which an agent reaches a waypoint is recorded, and these counts are then used to calculate the reward according to the classical count-based method; (2) we also take temporal distance^23,24^ as the basis for assigning rewards, with reward size determined by the environmental steps between the current observation and those in memory. Additionally, we encode the structure of the environment during exploration, and instead of constructing metric maps in previous studies^25–28^, we use a topological map to represent the state space. This space cognition can be used to gradually cover the environment by integrating observation sequences and as a planning module for navigation systems. Finally, we complement space cognition with a locomotion network that allows the agent to move between waypoints. Our contributions are as follows:

1. A novel navigation architecture that synchronizes learning exploration policy and encoding environmental structure.
2. An intrinsic motivation construct method that guides agents to spontaneously explore the environment and outperforms existing methods in terms of exploration efficiency.
3. Space topological cognition encodes the environmental structure by integrating observational sequences, which agents can use to deal with dynamic blockages without retraining or fine-tuning.

## Background

### Reinforcement learning foundation

Standard RL assumes that the agent interacts with the environment in many discrete time steps. At each time step $t$, the agent observes a state $s_{t} \left( {s \in S} \right)$ and chooses an action $a_{t} \left( {a \in A} \right)$ based on its policy $\pi$, where $\pi$ is a mapping from the state to the action. In return, the agent enters the next state $s_{t + 1}$ and receives a scalar reward $r_{t}$. This process is continuous until the maximum time steps of an episode or a terminal state are reached. The reward $R_{t} = \sum\nolimits_{k = 0}^{\infty } {\gamma^{k} r_{t + k} }$ is the accumulated return from time step $t$ with discount factor $\gamma \in \left( {0,1} \right]$. The target of the agent is to maximize the expected return from each state $s_{t}$, and there are two common methods to do this: value-based and policy-based methods.

The value function $V^{\pi } \left( \pi \right) = {\rm E}\left[ {R_{t} \left| {s_{t} = s} \right.} \right]$ is the expected return of the following policy $\pi$ from state $s$, and the more familiar action-value function $Q^{\pi } \left( {s,a} \right) = {\rm E}\left[ {R_{t} \left| {s_{t} = s,a} \right.} \right]$ is defined as the expected return of choosing action $a$ in state $s$ and the following policy $\pi$. In many RL approaches, the action-value function is represented by a function approximator, the famous one being Deep Q Network (DQN)^29^, which aims to approximate the optimal action-value function by a convolutional neural network (CNN). In contrast to the value-based method, the policy-based method directly parameterizes the policy $\pi \left( {a\left| {s;\theta } \right.} \right)$ and updates the parameter $\theta$ by the gradient ascent on ${\rm E}\left[ {R_{t} } \right]$. An example of such algorithms is REINFORCE^30^, which updates the policy parameter $\theta$ in the direction $\nabla_{\theta } \log \pi \left( {a_{t} \left| {s_{t} ;\theta } \right.} \right)$^31^.

**Figure 1:**
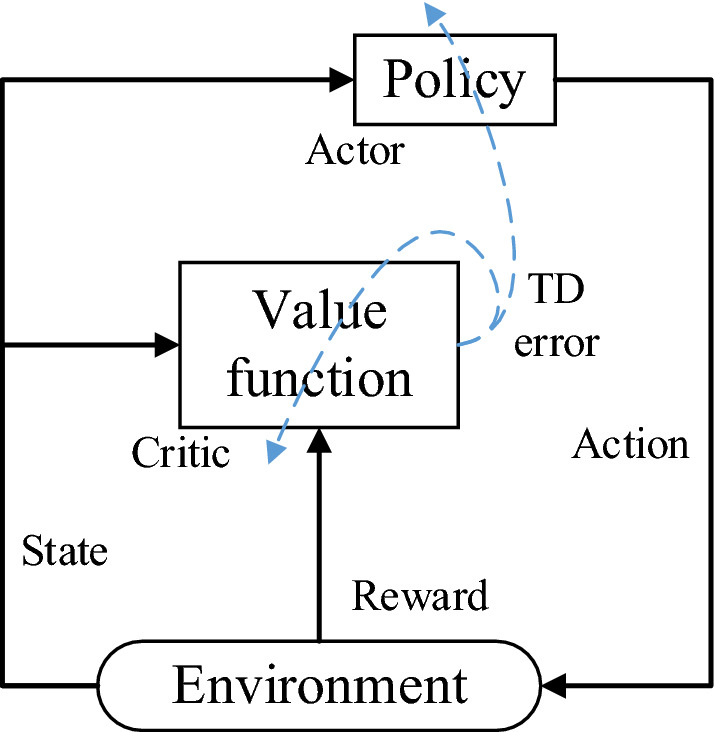
Flowchart of the AC algorithm.

As mentioned above, the value-based method and policy-based method have the same ultimate goal, but they use different methods to obtain policies, and each has its advantages and disadvantages. To combine the advantages of both, the actor-critic (AC)^32^ algorithm is proposed. Within the framework, the actor and critic are represented by policy $\pi$ and value function $V^{\pi } \left( {s_{t} } \right)$, respectively, and advantage estimation $A\left( {s_{t} ,a_{t} } \right) = Q\left( {s_{t} ,a} \right) - V\left( {s_{t} } \right)$ is used to scale the policy gradient. The operation diagram of the AC algorithm is shown in Fig. 1, which is an iterative optimization process.

### Asynchronous advantage actor-critic algorithm

The asynchronous advantage actor-critic (A3C)^7^ algorithm is an online RL learning method that maintains policy $\pi \left( {a_{t} \left| {s_{t} ;\theta } \right.} \right)$ and value function $V\left( {s_{t} ;\theta_{v} } \right)$ during interaction and relies on parallel actor-learners to provide accumulated updates. Similar to n-step Q-learning variant, A3C operates in the forward view and uses the same mixed n-step return to update the policy and value function after every $t_{\max }$ actions or when a terminal state is reached. The update is performed by the estimation of advantage function $A\left( {s_{t} ,a_{t} ;\theta ,\theta_{v} } \right)$ given by $R_{t} - V\left( {s_{t} ;\theta_{v} } \right)$, where $R_{t} = \sum\nolimits_{i = 0}^{k - 1} {\gamma^{i} r_{t + i} + \gamma^{k} V\left( {s_{t + k} ;\theta_{v} } \right)}$ and $k \in \left( {0,t_{\max } } \right]$. Furthermore, although the parameters $\theta$ of policy $\pi \left( {a_{t} \left| {s_{t} ;\theta } \right.} \right)$ and $\theta_{v}$ of value function $V\left( {s_{t} ;\theta_{v} } \right)$ are computed and updated separately, sharing some parameters and adding entropy regularization terms have been shown to help in learning control policies.

**Figure 2:**
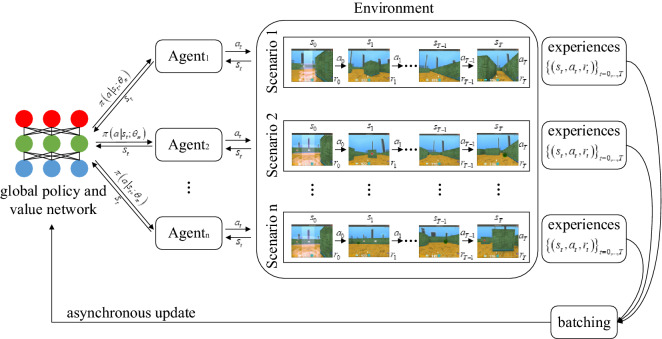
Flowchart of the A3C algorithm.

In the A3C algorithm, each agent interacts with the environment independently. Due to the random initialization of parameters, the observed state, actions taken and rewards achieved are different between parallel agents, as shown in Fig. 2, thus enabling asynchronous update and reducing the relevance of the training samples. Similar to other nonsynchronous methods, the loss function of the policy and value function are calculated by Eqs. (1) and (2), respectively:

1 $$f_{\pi } \left( \theta \right) = \log \pi \left( {a_{t} \left| {s_{t} ;\theta } \right.} \right)\left( {R_{t} - V\left( {s_{t} ;\theta_{v} } \right)} \right)$$

2 $$f_{v} \left( \theta \right) = \left( {R_{t} - V\left( {s_{t} ;\theta_{v} } \right)} \right)^{2}$$

The losses of each agent are collected and the global network is updated using the standard non-centered RMSProp, as shown in Eqs. (3) and (4). After each update, the global network transmits the policy and value function to each agent.

3 $$g = \alpha g + \left( {1 - \alpha } \right)\nabla \theta^{2}$$

4 $$\theta \leftarrow \theta - {{\eta \nabla \theta } \mathord{\left/ {\vphantom {{\eta \nabla \theta } {\sqrt {g + \varepsilon } }}} \right. \kern-\nulldelimiterspace} {\sqrt {g + \varepsilon } }}$$

where $g$ is the moving average of the elementwise squared gradients, $0 \le \alpha \le 1$ is a hyperparameter, $\eta$ is the learning rate and $\varepsilon$ is a constant added to maintain numerical stability.

### Nav A3C model

A prominent application of the A3C algorithm in the navigation field is Nav A3C^3^, which is an end-to-end framework that incorporates multiple objectives. Similar to the A3C algorithm, Nav A3C maximizes accumulated return through actor-critic architecture and uses policy $\pi \left( {a_{t} \left| {s_{t} ;\theta } \right.} \right)$ and value function $V\left( {s_{t} ;\theta_{v} } \right)$ to select actions.

**Figure 3:**
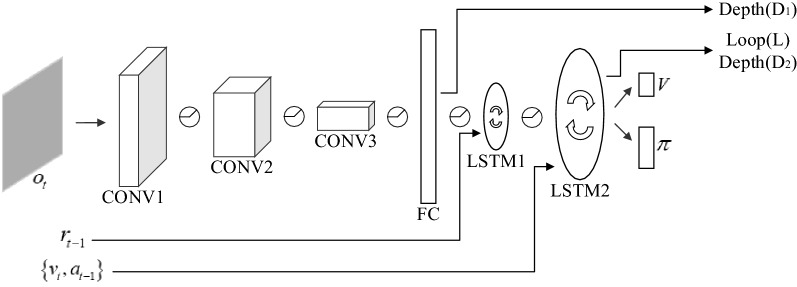
The Nav A3C model.

Details about the architecture of Nav A3C are shown in Fig. 3. Its encoder is a three-layer convolutional neural network, and to address the memory requirement, Nav A3C employs a stacked LSTM after that. The inputs to this model include: the agent observation $o_{t} \in R^{3 \times W \times H}$(where $W$ and $H$ are the width and height of the image), the velocity $v_{t} \in R^{6}$, the previous action $a_{t - 1} \in R^{{N_{A} }}$ and the previous reward $r_{t - 1} \in R$. Inside the model, the first LSTM layer receives the reward, and the velocity and previously selected action are fed directly to the second recurrent layer. The policy and value functions share all intermediate representations, and each of them is computed by a linear layer. In addition, two auxiliary tasks are used in the Nav A3C model, which we also illustrate in Fig. 3: D_1_ and D_2_ are designed to use additional losses to provide depth information about the environment, and the loop closure classification task (L). In the Nav A3C + D_2_(D_1_)L model, which incorporates auxiliary tasks, the agent is trained by applying a weighted sum of the gradients coming from A3C, the gradients from depth prediction and the gradients from loop closure.

## Navigation method

In the following sections, we introduce all the components of our method. The first section describes how to create rewards for the agent and use them to guide exploration behaviour. Then, the method for encoding the environment with episode memory as inputs is presented, and finally, the target-driven navigation approach is illustrated.

### Learning exploration policy

#### Temporal correlation network

The temporal correlation network (TC-network, $\phi_{TC}$) is trained to compute the temporal distance between observations, which is essential for creating rewards and encoding environments. Additionally, the visual perception tasks, including agent location and target detection, rely on this network.

**Figure 4:**
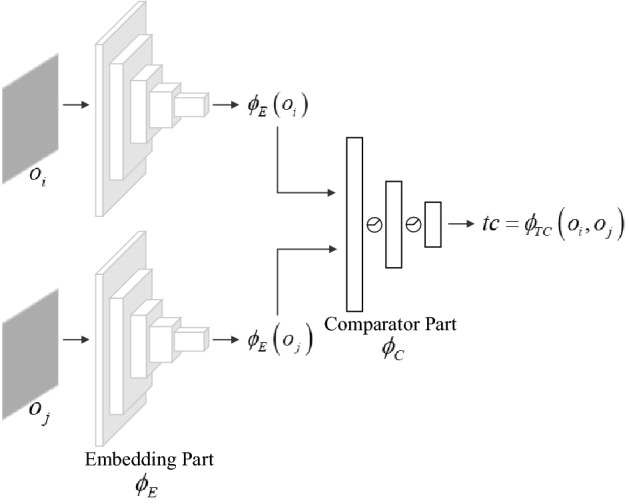
The TC-network model.

Conceptually, we view the TC-network as a classification task: the network is trained to assign high similarity to temporally close observation pairs and low similarity to temporally distant observation pairs. The architecture of the TC-network is shown in Fig. 4, which consists of two parts: an embedding part $\phi_{E}$, which is based on ResNet-18^33^ and used to encode visual input, and a comparator part $\phi_{C}$ that takes the features as input, and outputs the temporal correlation coefficient $tc$ between observations (such as $o_{i}$ and $o_{j}$):

5 $$tc = \phi_{TC} \left( {o_{i} ,o_{j} } \right) = \phi_{C} \left( {\phi_{E} \left( {o_{i} } \right),\phi_{E} \left( {o_{j} } \right)} \right)$$

The training samples of the TC-network are triples $\left\langle {o_{i} ,o_{i + k} ,y_{ik} } \right\rangle$, which consist of two observations and a binary label. These observations are considered close $\left( {y_{ik} = 1} \right)$ if they are separated by at most $k$ steps. Negative examples are pairs where the two observations are separated by at least $M \cdot k$ steps, and the hyperparameter $M$ is necessary to create a gap between positive and negative examples. Finally, the network is trained with logistic regression losses to output the probability of positive classes.

#### Formulate reward function

Everyone knows that agents cannot take near-optimal actions until they fully explore their environment or construct a cognitive model of the world^34^, but the basic question is how to obtain an efficient exploration policy. Relying on simple action entropy maximization as a source of exploration behaviour is difficult in complex environments, and annotating each environment with a hand-designed dense reward is not scalable. One available alternative is curiosity-driven exploration^35^, which is inspired by biological mechanisms and uses intrinsic motivation to guide exploration. Across different fields, many theorists have suggested the pattern of intrinsic motivation, including empowerment^36^, surprise^37^, and novelty^38^. The way we make up the reward function is based on novelty theory, which shows the animal can reward itself for something novel. Furthermore, our reward function consists of two novelty reward types, both of which are related to episodic memory.

The first part of our reward function is calculated based on a count-based method, and for those models that use the same approach, the novelty of a state-action pair is derived from the number of times the agent visits the pair. Such approaches require an enumerable environmental representation to prevent dimensional explosion problems, which is why the count-based method is not practical for high-dimensional state spaces since most states occur only once. Our approach discretizes the state space by TC-network $\phi_{TC} :S \to W$ and uses waypoints $o^{w} \left( {o^{w} \in W} \right)$ to represent the environment. States are mapped to waypoints, so their occurrences can be counted by the corresponding waypoints. These counts are then used to calculate the novelty reward according to classical count-based exploration theory, and such reward $r^{cb} :S \to R$ is defined as:

6 $$r^{cb} \left( {o^{c} ,o^{w} } \right) = \frac{\alpha }{{\sqrt {n\left( {\phi_{TC} \left( {o^{c} ,o^{w} } \right)} \right)} }}$$

where $\alpha \in R_{ \ge 0}$ is the reward coefficient, $o^{c}$ is the current observation, and $o^{w}$ is the waypoint. For every mapping $o^{c} \to o^{w} \left( {o^{w} \in W} \right)$ found, the corresponding $n\left( {\phi_{TC} \left( {o^{c} ,o^{w} } \right)} \right)$ is increased by one. You might be thinking that the count-based method can effectively calculate the novelty reward for each state that has mappings. However, if the mapping does not exist, in other words, the current observation is in the unexplored part of the environment, then how is the reward calculated? That is the next question we want to address.

As described above, animals can reward themselves when they see something novel, but the size of the reward varies with the effort put in by the agent. This idea can be formalized as giving rewards to observations outside the already explored part of the environment, and the magnitude of the reward is proportional to the shortest temporal distance between the current observation and the waypoints. Therefore, the other part of our reward function $r^{td} :S \to R$ is defined as:

7 $$r^{td} \left( {o^{c} ,o^{w} } \right) = \mathop {\min }\limits_{{o^{w} \in W}} \left\{ {\frac{\beta }{{\phi_{TC} \left( {o^{c} ,o^{w} } \right)}}} \right\}$$

where $\beta \in R_{ \ge 0}$ is the reward coefficient, $o^{c}$ is the current observation, and $o^{w} \left( {o^{w} \in W} \right)$ is the waypoint stored in memory. The reward function $r^{i} \left( {o^{c} ,o^{w} } \right)$ is defined as the sum of the two novelty reward types:

8 $$r^{i} \left( {o^{c} ,o^{w} } \right) = r^{cb} \left( {o^{c} ,o^{w} } \right) + r^{td} \left( {o^{c} ,o^{w} } \right)$$

**Figure 5:**
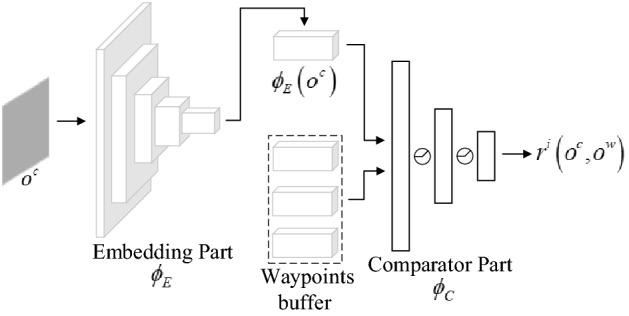
The reward calculation process.

The reward calculation process is shown in Fig. 5. To determine the novelty rewards of current observations, we must keep track of what has been explored in the environment. Waypoints buffer provides a good choice, they store instances of the past and can update in time as regions are explored.

#### Learning model

**Figure 6:**
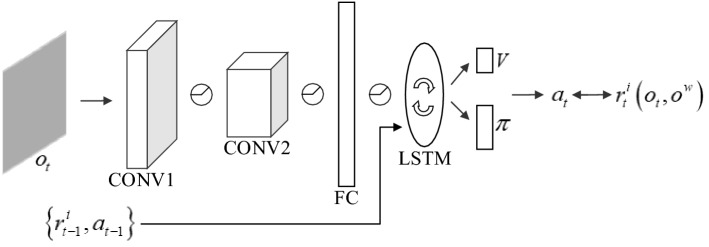
The learning model.

DRL provides an effective framework to enable agents to acquire a control policy for a particular task^39^, and in our method, it is used as the basic method for learning exploration policy. Our learning model is shown in Fig. 6, with an architecture that references the Nav A3C model and makes some adjustments to fit the exploration task. One of the most obvious changes occurs in the structure of the encoder and the memory unit. Because our method does not utilize auxiliary tasks, it does not require a stacked LSTM network to store additional environmental information. The 1-layer LSTM can satisfy the memory requirement, and the convolutional encoder can be relaxed from 3 to 2 layers. To further reduce the training complexity, our convolutional layers output 16 and 32 features, respectively, instead of 32 and 64 for the Nav A3C model. The input to this model includes: the observation $o_{t} \in R^{3 \times W \times H}$(where $W$ and $H$ are the width and height of the image), the previous action $a_{t - 1} \in R^{\left| A \right|}$, and the previous reward $r_{t - 1}^{i} \in R$. At every time step $t$, action $a_{t}$ is selected to maximize reward $r_{t}^{i}$. It should be noted that the reward $r_{t}^{i}$ does not include any reward from the environment, except for these two types of novelty rewards. We use the A3C algorithm with n-step look-ahead values to tune the policy $\pi \left( {a_{t} |s_{t} ;\theta } \right)$ and value function $V\left( {s_{t} ;\theta } \right)$ and a regularization penalty for entropy to prevent premature convergence. During the training process, many agent instances interact in parallel with many environmental instances.

### Space topological cognition

Episodic memory is one of the sources of our cognition of the world and the basis for animals’ encoding their environment. However, encoding the environment using these observation sequences remains a problem due to their complexity and redundancy. Inspired by the grid cells^40^ found in the rat brain, we proposed the concept of waypoints. Waypoints are a special class of observations, each of which can represent a state space within a temporal distance. Relying on the TC-network, we can find waypoints in episodic memory and use them to encode the environment during exploration, and the encoding process can be divided into two stages.

**Figure 7:**
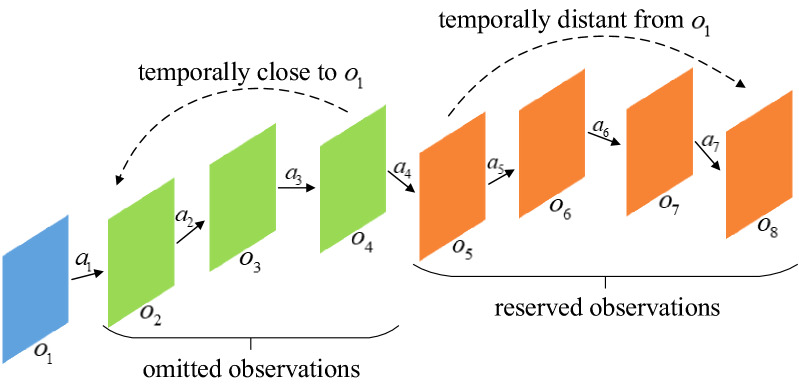
Schematic diagram depicting a simplified version of the observation sequence.

In the initial stage, we need to encode the exploration sequence, as there are no waypoints stored in memory. Assume the agent lasts for $T$ time steps in an episode and produces an observation sequence $\left( {o_{1} ,o_{2} ,...,o_{T} } \right)$. Taking the first elimination of redundant observations as an example, the temporal correlation coefficient between $o_{1}$ and the others in the sequence is obtained via the TC-network:

9 $$tc^{1} = \phi_{TC} \left( {o_{1} ,o_{i} } \right)$$

where $tc^{1}$ represents the temporal correlation coefficient of the first iteration and $i \in \left[ {2,T} \right]$. According to the threshold *tc*^*th*^, the observations adjacent to $o_{1}$ are omitted, and the simplified diagram is shown in Fig. 7. This is the first iteration where all the retained observations cannot be mapped to observation $o_{1}$, so $o_{1}$ is the first waypoint $o_{1}^{w}$ stored in memory. Then, the sequence is continuously simplified with the retained observations and in the same way until the last observation.

It is worth noting that our method follows the order of observation, and the waypoints within the memory are stored incrementally and connected in theory. However, the agent’s movement is realized by the locomotion network, and the connection between waypoints should be considered to determine whether it applies to the locomotion network. In two cases, we connect waypoints with an edge: if the corresponding waypoints are reachable according to the judgement of the TC-network, or if they are within certain time steps:

10 $$e = 1 \Leftrightarrow \phi_{TC} \left( {o_{i}^{w} ,o_{j}^{w} } \right) > tc^{re} \vee \left| {i - j} \right| \le k$$

where $e$ is the connection relationship between waypoints, $o_{i}^{w}$ and $o_{j}^{w}$ are waypoints, and $tc^{re} \in \left( {0.5,tc^{th} } \right)$ is the threshold of reachability. The first edge type corresponds to temporal distance, while the second type corresponds to natural spatial adjacency between locations, and both connection types are acceptable for the locomotion network.

**Figure 8:**
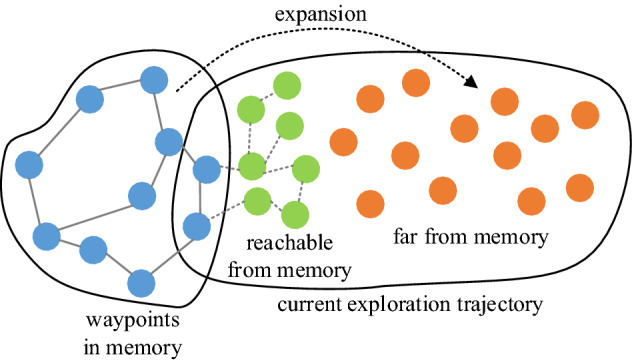
Schematic diagram of cognitive expansion.

In the extension stage, as shown in Fig. 8, we need to continuously discover waypoints in the current exploration trajectory and use them to extend the space cognition.

Therefore, each observation in the current sequence $\left( {o_{1} ,o_{2} ,...,o_{T} } \right)^{c}$ needs to be compared with the waypoints to obtain the temporal correlation coefficient between them:

11 $$tc^{c} = \phi_{TC} \left( {o_{i}^{c} ,o_{j}^{w} } \right)$$

where $tc^{c}$ represents the temporal correlation coefficient between the current sequence and waypoints, $o_{i}^{c} \left( {i \in \left[ {1,T} \right]} \right)$ is the observation in the current sequence, and $o_{j}^{w} \left( {j \in \left[ {1,n} \right]} \right)$ is the waypoint stored in memory. If all observations in the current sequence can be represented by waypoints, there is no need to update the space cognition. Conversely, if there are observations that cannot be mapped by waypoints, then these observations become new waypoints and are added to the memory. At this point, the edges connecting the new waypoints need to be created:

12 $$e = \left\{ {\begin{array}{*{20}c} 1 & {\phi_{TC} \left( {o_{i - 1}^{c} ,o_{j}^{w} } \right) > tc^{re} } \\ 0 & {i = 1} \\ \end{array} } \right.$$

where $o_{i - 1}^{c}$ is the previous observation of $o_{i}^{c}$, $o_{i}^{c} \left( {i \in \left[ {2,T} \right]} \right)$ is the added waypoint from the current sequence, and $o_{j}^{w} \left( {j \in \left[ {1,n} \right]} \right)$ is a waypoint in the memory.

### Navigation implementation

#### Locomotion network

The purpose of the locomotion network (L-network, $\phi_{L}$) is to assist the agent in completing the motion between waypoints, which maps a pair of observations $\left( {o_{i} ,o_{j} } \right)$ and produces probabilities $P\left( {o_{i} ,o_{j} } \right) \in R^{\left| A \right|}$.

Since the input to the L-network are images, the first thing that comes to our mind is the use of inter-frame difference^41^ to predict actions, whose salient feature is its real-time nature. However, this approach always runs into trouble in two areas: limited prediction accuracy and easy distraction. To overcome these problems, we use feature space rather than pixel-level inputs to predict actions, and objects encoded by the L-network can be classified into three types: (1) objects that can be influenced by the agent’s actions, (2) objects that cannot be influenced by the agent’s actions but whose actions can influence the agent, and (3) objects that are completely unrelated to the agent’s actions. A good feature space for predicting actions should be closely related to (1) and (2) and not interfered with by (3). The latter is because if there is a variation that is unconsidered for the agent’s actions, then the agent has no incentive to know about it.

**Figure 9:**
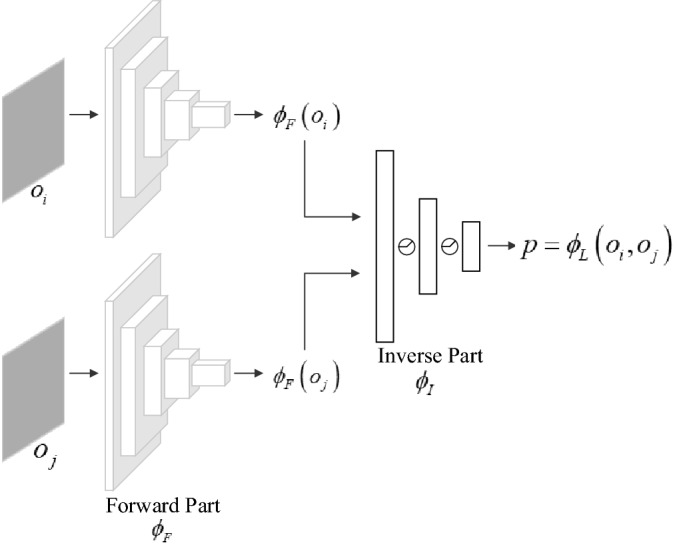
The L-network model.

In contrast to manually designed features, we use a deep neural network (DNN)^42^ to generate features automatically. The L-network model is shown in Fig. 9, which is an end-to-end framework with a forward-inverse structure. One advantage of this framework is that actions are not separated from features but learned together, thus ensuring that there is no incentive for features to encode any objects that cannot influence or are not influenced by the agent’s actions. Inside the model, the forward part $\phi_{F}$ is a deep convolutional encoder based on ResNet-18 that calculates the raw observations $\left( {o_{i} ,o_{j} } \right)$ as feature vectors; the inverse part $\phi_{I}$ takes the features as input and produces the probabilities of actions:

13 $$P = \phi_{L} \left( {o_{i} ,o_{j} } \right) = \phi_{I} \left( {\phi_{F} \left( {o_{i} } \right),\phi_{F} \left( {o_{j} } \right)} \right)$$

The training samples of the L-network are similar to those of the TC-network in that it contains a pair of observations separated by $k$ steps and an action: $\left( {\left( {o_{i} ,o_{i + k} } \right),a_{{o_{i} }} } \right)$, where the action corresponds to the first observation. Training the L-network amount to the learning function $\phi_{L}$ is defined as:

14 $${\mathop a\limits^{ \wedge}}_{{o_{i} }} \Leftrightarrow P = \phi_{L} \left( {o_{i} ,o_{i + k} ;\theta_{L} } \right)$$

where $\hat{a}_{{o_{i} }}$ is the prediction of action $a_{{o_{i} }}$, and the neural network parameters $\theta_{L}$ are trained to optimize $\mathop {\min }\limits_{{\theta_{L} }} loss\left( {\hat{a}_{{o_{i} }} ,a_{{o_{i} }} } \right)$ in a supervised fashion, with a softmax output layer and the cross-entropy loss.

#### Goal reaching process

**Figure 10:**
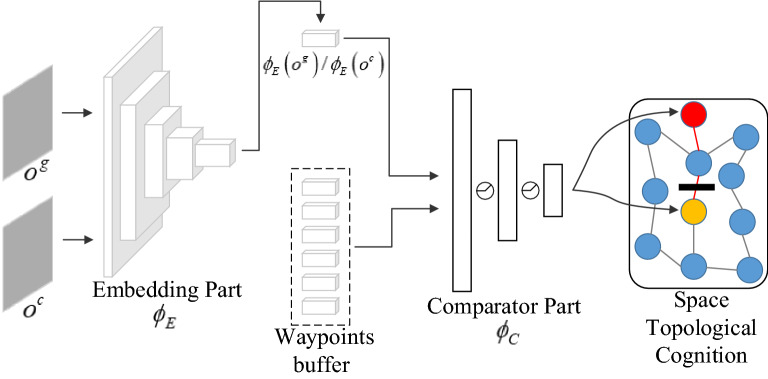
The location method.

The navigational tasks are performed in an episodic setting where each episode lasts for fixed time steps. In an episode, the agent uses the space topological cognition constructed during exploration to find an available path to achieve the goal, so we first need to know where the path begins and ends. The location method is shown in Fig. 10. Relying on the TC-network and environmental memory, we can use the goal images $o^{g}$ collected during the training process to obtain the location of the goal (red circle) and use the current observation $o^{c}$ to locate the agent (yellow circle).

After obtaining the locations of the agent and the goal, we use Dijkstra's algorithm^43^ to find the shortest path between waypoints $o_{s}^{w}$ and $o_{g}^{w}$, marked in red in the topological cognition (Fig. 10) and denoted by Formula (15):

15 $$\left\langle {o_{s}^{w} ,o_{1}^{w} ,...,o_{g}^{w} } \right\rangle ,o_{s}^{w} = o^{c} ,o_{g}^{w} = o^{g}$$

**Figure 11:**
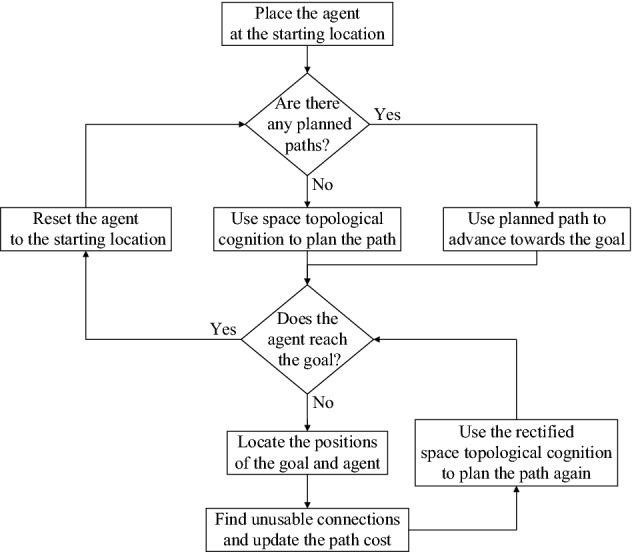
Flowchart of the procedure for reaching the goal.

However, this is not the end of the story. Since space topological cognition is learned in barrier-free environments, the planned paths produced by it are only applicable in non-blocking environments. When there are blockages in the state space, especially a blockage presents in the navigation path (the black blockage in Fig. 10), the agent will stay in front of the blockage. To deal with this situation, space topological cognition must be updated in time according to changes in the environment. In our method, this adjustment is achieved by changing the path cost of unavailable connections between waypoints. During navigation, if the goal is not reached within a certain number of time steps, it proves that there are blockages in the planned path. We need to find the connections that traverse blockages and set their path cost to infinity, thus ensuring that unavailable connections cannot be used for path planning. The new path needs to be replanned based on the revised space cognition. Since waypoints are interconnected and there may be multiple blockages in the environment, reaching the goal is a dynamic process, the flow of which is shown in Fig. 11.

## Experiment

In this section, we evaluate the performance of our method on exploration and goal reaching tasks and compare it to relevant baselines.

### Experiment setup

#### Experimental environment

**Figure 12:**
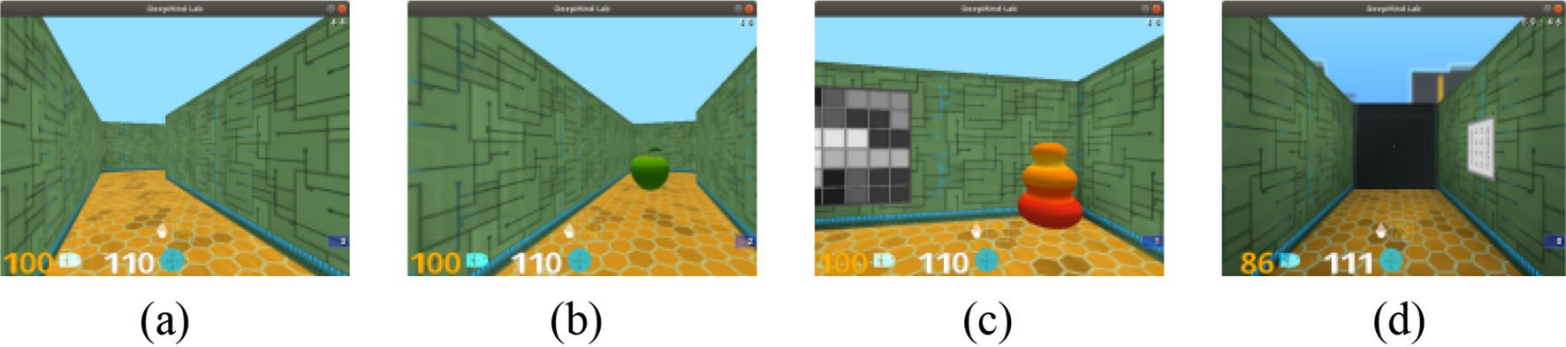
Simulation environment. (**a**) Go forwards. (**b**) Apple. (**c**) Goal. (**d**) Door.

We test our approach and relevant baselines in multiple mazes in DMlab^44^, and an illustration of an agent navigating towards a goal in the environment is shown in Fig. 12. In this 3D simulation environment, the agent perceives the environment from a first-person perspective and has access to additional environmental information such as inertial information and local depth information. The action space is discrete while allowing fine control and includes 6 actions: move forwards/backwards, turn left/right, turn left/right + move forwards. The environment runs at 60 frames per second and extrinsic rewards are achieved by reaching apple (worth + 1 point) and goal (worth + 10 points) in the environment. If the goal is reached, the agent is respawned to a new start location, and the episode does not end until a fixed number of time steps. The software environment for this experiment is Ubuntu 18.04, and the hardware environment is a DELL T7920 workstation with 64 GB RAM, an Intel Xeon Gold 5118 CPU and two Nvidia RTX 2080TI graphics cards. All programs in the experiment were implemented in the Python language.

#### Baselines

In experiments in which agents were guided to produce exploration behaviour, we compared our method to a baseline set that also had intrinsic motivation as a motivator for exploration. The simplest baseline is the basic RL algorithm trust region policy optimization (TRPO)^45^, which relies on the heuristic $\varepsilon { - }greedy$ method to encourage exploration. Then, we use VIME^19^ as a comparator, which is based on a Bayesian neural network (BNN), to perceive dynamic changes in the environment and obtain an exploration policy by maximizing this information gain. The third baseline is a classifier-centric approach called EX^2^^20^, which explores novelty detection relying exclusively on a discriminatively trained exemplar model. Finally, as a sanity check, we also reproduce the state-of-the-art curiosity method ICM^21^, which makes predictions in the feature space and formulates curiosity as the error of its actions.

In goal reaching experiments, we take three models equipped with the DRL framework as baselines. One is the well-known feedforward model DQN^29^, the other is the recurrent model Deep Recurrent Q Network (DRQN)^46^, and the last is Nav A3C^3^, which has been mentioned before. In addition, the enhanced version Nav A3C + D_2_L, which jointly learns goal-driven behaviour with auxiliary tasks, is reported in the experiments.

#### Model implementation

The architecture details of our learning model are as follows. It has two convolutional layers: the first layer has $8 \times 8$ filters applied with stride $4 \times 4$ and 16 feature maps, while the second layer has $4 \times 4$ filters with stride $2 \times 2$ and 32 feature maps. This is followed by a fully-connected layer with 256 units, and all three layers are followed by a ReLU nonlinearity unit. After that, an LSTM layer with 256 units is used to take the CNN-encoded observation, previous action and reward as input, and the policy and value function are linear projections of the LSTM layer output.

For the TC-network and L-network, their inputs are two observations, each of which is processed by the ResNet-18 encoder and produces a 512-dimensional feature vector. The TC-network first concatenates these features and then puts them in a fully connected network with 4 hidden layers, each with 512 units and a ReLU nonlinearity unit, to predict whether the two observations are adjacent to each other. Likewise, the L-network processes these features together after concatenating them in series. The fully-connected part consists of 2 hidden layers, each with 512 units and a ReLU nonlinearity unit, and a softmax layer with 6 outputs, which correspond to all available actions of the agent.

#### Hyperparameters

During the exploration, we choose the commonly used A3C algorithm as the basic RL approach and take $84 \times 84$ RGB observations at 3 frame intervals (4 repetitions of each action) as input. There are 8 workers equipped with a non-centered RMSProp that interact with the environment in parallel. The learning rates are sampled from a log-uniform distribution between 0.0001 and 0.005, and the entropy costs are sampled from a log-uniform distribution between 0.0005 and 0.01.

The inputs to the TC-network and L-network are two RGB images at resolution $160 \times 120$ pixels, and all the training data are generated by agents themselves. During the training process, we randomly sample a mini-batch of 64 observation pairs from the replay buffer $B$ and perform an update using the Adam optimizer^47^ with learning rate $\lambda = 0.0001$.

### Parameter selection experiment

**Figure 13:**
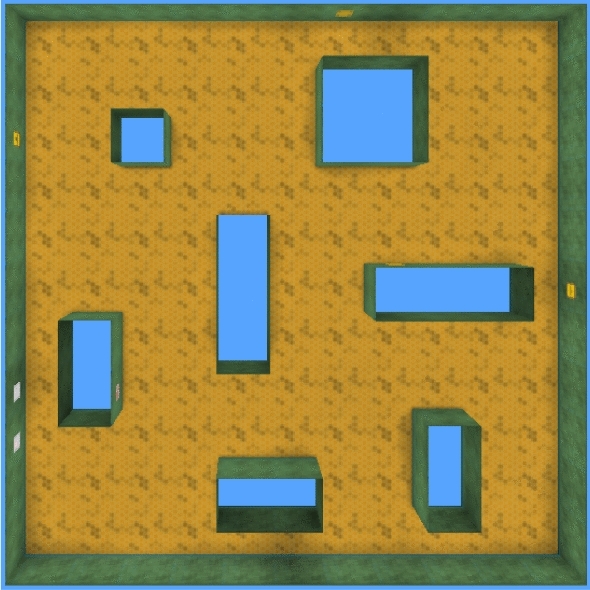
Parameter selection environment.

We are interested in agents that can spontaneously explore and encode the environment, but some parameters need to be set in advance before testing the performance of our approach. These parameters are related to two aspects, the training details of the TC-network and L-network, and the key factors of the reward function, and we identify them in the maze shown in Fig. 13.

#### Sample separation parameter

In training the TC-network, a threshold $k$ is needed to distinguish between positive and negative sample pairs, and the training samples of the L-network are also distinguished by this value. Therefore, we conduct an experiment by varying $k$ from 1 to 10 and show its effects on these networks. The training results of the TC-network and L-network (1.5 M interaction quantity) are averaged over the top 5 random hyperparameters, and the proportion of waypoints is calculated based on the corresponding TC-network and 30 random observation sequences. Table 1 shows that the training effect of the TC-network is closely related to the difference between positive and negative samples. At the beginning, the accuracy of the TC-network is low due to the small differences between samples. Then, the prediction ability increases with $k$, but the accuracy decreases again when the threshold is greater than a value. Compared with the TC-network, the performance of the L-network decreases continuously with the growth of $k$, especially after $k > 4$. Finally, the number of waypoints decreases as $k$ increases, but when $k$ is greater than a value, as we mentioned before, the predictive power of the TC-network reaches a bottleneck, leading to an increase in the number of waypoints.

**Table 1:** The experimental results of the sample separation parameter.

Threshold $k$	TC-network (%)	L-network (%)	Waypoints proportion (%)
1	88.68	95.74	31.25
2	91.53	95.26	22.34
3	93.06	93.42	16.27
4	92.32	91.04	12.51
5	90.87	86.35	11.63
7	86.59	80.73	12.48
10	81.93	75.68	12.65

The experimental results create a dilemma because the TC-network and L-network are key to the encoding environment and navigation, and we must keep them in good condition. However, we also support storing as few waypoints as possible during the interaction. After comprehensive consideration, we choose data separated by 4 time steps as training samples.

#### Interaction volume parameter

In addition to the threshold $k$, the amount of interaction with the environment is another important parameter in the pretraining phase. In our method, the complexity of the sample consists of two parts: pretraining and online learning. Exploration behaviour is performed through online learning without concern for sample size, but the pretraining effect is related to the number of samples. Table 2 shows the relationship between the interaction volume and the network performance, and the results are averaged over the top 5 random hyperparameters. As shown in Table 2, the accuracy of the TC-network increases with the expansion of the training data and decreases when the network is in an overfitting state. Similarly, the prediction accuracy of the L-network improves as the interaction volume increases, but the percentage of growth gradually decreases. To train efficiently and maintain the good performance of both networks, the number of pretrained interactions is set as 2.5 M.

**Table 2:** The experimental results of the interaction volume parameter.

Sample size	TC-network (%)	L-network (%)
300 K	80.35	82.91
500 K	82.42	86.57
1 M	87.95	90.83
2.5 M	92.63	93.78
5 M	91.02	93.94

In summary, the TC-network and L-network can learn useful controllers based on the trajectories of randomly acting agents and use them for visual perception tasks and short-range navigation. However, since all samples in the pretraining phase are from the same environment, this inevitably leads to a lack of generality in these networks. Therefore, during the subsequent exploration, we collect data from different environments to train these networks twice.

#### Reward function parameter

**Figure 14:**
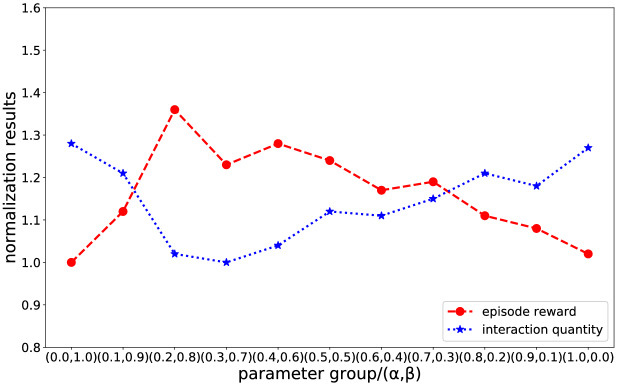
Experimental results on the reward function parameter.

Our reward function $r^{i}$ is an augmented reward that includes two types of novelty rewards. To weigh the effects between them, we test the effects of different parameter sets that are set $\alpha + \beta \equiv 1$ and sampled within the same interval (0.1) and show two main results: the episode reward (novelty rewards achieved by the agent within 1800 time steps) and the number of interactions required to encode the environment. The results are averaged over the top 5 random hyperparameters and summarized in Fig. 14 after normalization of the data (the lowest result is taken as the criterion). As shown in Fig. 14, relying on a type of novelty reward, where $\alpha ,\beta = \left( {0.0,1.0} \right)$ or $\alpha ,\beta = \left( {1.0,0.0} \right)$, the agent can generate various exploration behaviours. However, their exploration is less efficient than agents who use both novelty rewards, which is why these agents require more interaction to encode the environment. Additionally, we can explain the experimental results in terms of the composition of the reward function. Our reward function consists of two parts, each of which focuses on one direction: (1) the count-based method focuses on novelty rewards for already explored environments and encourages the agent to reach seldom visited waypoints, (2) the temporal distance method focuses on calculating novelty rewards for unexplored state spaces and tries to push the agent to distant places. Therefore, it is useful to use these two rewards to guide the exploration.

Among all the agents, the one equipped with parameter sets $\alpha ,\beta = \left( {0.2,0.8} \right)$ shows the best exploration efficiency and requires less interaction to encode the environment, so we choose $\alpha = 0.2$ and $\beta { = }0.8$ in the following experiment. In addition, unlike the pretraining stage, agents no longer act randomly but learn the exploration policy in the environment (Fig. 13), which is the basis for fine-tuning in the other mazes.

### Exploration method experiment

**Figure 15:**
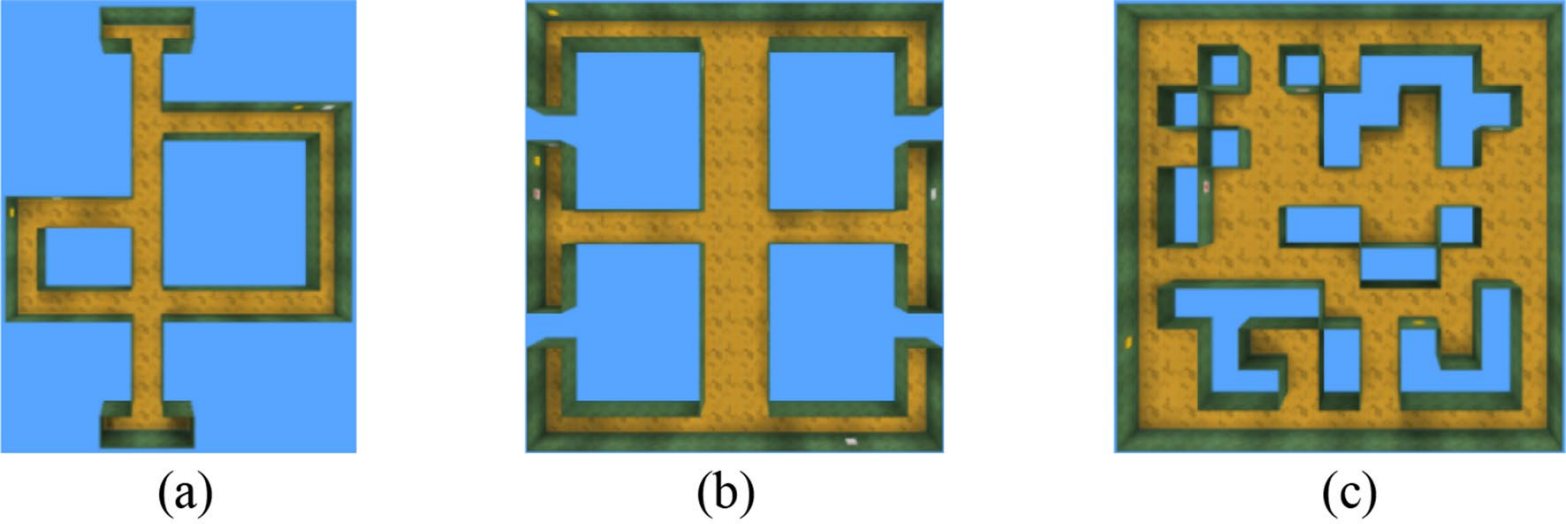
Top-down view of testing mazes. (**a**) Maze-1. (**b**) Maze-2. (**c**) Maze-3.

This experiment aims to quantitatively evaluate the exploration performance of different learning approaches and training patterns and to illustrate their effects on the efficiency of the encoding environment. The test environments are shown in Fig. 15, where Maze-1 and Maze-2 are inspired by rodent spatial cognition experiments; the former consists of three paths of different lengths, and the latter consists of a central corridor and six arms. Maze-3 is a common maze that includes different obstacles and multiple paths. There are no extrinsic rewards (such as a goal or fruit) in these mazes.

The performance of each method is evaluated by a uniform count-based reward that is calculated based on the area explored by the agent in an episode. The learning process is presented as an episodic reward/training step diagram, where the agent must explore the environment as much as possible within a time limit of 7200 steps (equivalent to 2 min). At the end of each episode, the agent is respawned into a new location and has to explore the environment again.

#### Learning exploration from scratch

In our first set of experiments, all exploration policies are learned from scratch. Since the TC-network and L-network require an additional 2.5 M data to complete the pretraining, for a fair comparison, we shift the curves for our method by the number of environmental steps taken to train these networks.

**Figure 16:**
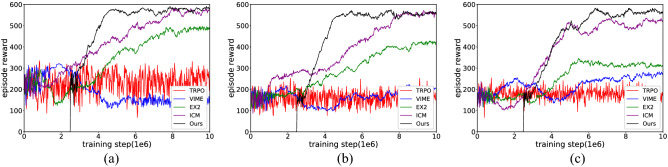
The experimental results of learning exploration from scratch. (**a**) Maze-1 experiment results. (**b**) Maze-2 experiment results. (**c**) Maze-3 experiment results.

The training curves (averaged over the top 5 random hyperparameters) are shown in Fig. 16. Through the analysis, we draw some conclusions. First, the VIME method achieves good results on simple and clean images in the Atari game but performs poorly in all test mazes. This is because BNN is not sufficient for constructing a dynamic model based on the first-person perspective, so the agents only perform some reactive behaviours during the learning process. The worst case occurs in Maze-1, where this generative model explores areas even smaller than the random-action agent. Second, EX^2^ is more suitable for challenging image-based environments than VIME, which generates coherent exploration behaviour and guides the agent to reach alcoves of the end in Maze-2. However, EX^2^ requires a large number of interactions to train the exemplar model, leading it to obtain rewards below 300 in the early training phase. Because of the limited capability of the classifier, as the structure of the maze becomes more complex, more regions are ignored by the agent because they are not rewarded with the novelty they deserve. Finally, for both ICM and our method, they achieved exploration policy greatly exceeding the prior exploration techniques, which proves that both methods are suitable for high-dimensional continuous state spaces. The efficiency of exploration between them is more evident in Maze-3. Because this environment includes many obstacles and hidden areas, relying on prediction errors alone tends to produce dead spots for exploration. Our method, in contrast, generates intrinsic rewards based on episode memory that can drive the agent to explore every corner of the environment. It is also important to note that although the final rewards obtained by ICM and our method in Maze-1 and Maze-2 are almost identical, our method can push the agent to reach distant states and discover more areas through the same interaction.

Table 3 collects the results averaged over the 5 best performances in the learning process. As seen in Table 3, the rewards achieved and the maximum exploration rate increased as the exploration efficiency increased, and the detailed results for each method are described below. To our surprise, the basic exploration method TRPO, whose behaviour relies on random actions, still covers half of Maze-1 and obtains almost equal rewards to the VIME method in Maze-2. For the second baseline, because the generative model lacks proper inference about environmental dynamics, it is unable to consciously explore the environment and act like purposeless people. The following methods show better performance in high-dimensional visual environments, with EX^2^ achieving at least 70% coverage in the first two mazes and allowing the agent to reach more than 50% of the area in Maze-3; ICM guides the agent to gain complete cognition of Maze-1 and Maze-2, but it requires a large number of interactions to stabilize the exploration behaviour, and this reasonable policy is not guaranteed in Maze-3. Compared with previous methods, our approach maintains an effective exploration policy in each test maze and uses it to drive agents to cover the environment. Additionally, our method outperforms other methods both in terms of exploration efficiency and the quantity of training data required for policy convergence, a phenomenon evident in Maze-1 and Maze-2.

**Table 3:** The experimental results of learning exploration from scratch.

Environment	Method	Reward	The maximum exploration ratio within an episode (%)	The interaction required to encode environment (training step/1e6)
Maze-1	TRPO	327.36	55.29	$\infty$
	VIME	321.14	53.58	$\infty$
	EX^2^	489.27	82.43	$\infty$
	ICM	584.59	100.00	7.93
	Ours	586.32	100.00	4.72
Maze-2	TRPO	232.47	41.02	$\infty$
	VIME	228.34	39.98	$\infty$
	EX^2^	425.73	74.56	$\infty$
	ICM	567.28	100.00	8.07
	Ours	571.87	100.00	5.15
Maze-3	TRPO	243.49	41.73	$\infty$
	VIME	276.54	47.82	$\infty$
	EX^2^	339.62	58.35	$\infty$
	ICM	532.27	91.64	$\infty$
	Ours	579.65	100.00	6.54

In addition, as seen in Table 4 (the results are averaged over 3 times), the accuracy of the TC-network and L-network completed in the pretraining stage decreases rapidly in the new test environments. If we insist on not making any adjustments to them, the performance of subsequent encoding and navigation will definitely suffer. We also find that training these networks for each maze is not a wise choice, because this targeted method, while yielding better predictive power, increases the cost of pretraining exponentially and pushes back the process of encoding the environment. Therefore, we train the TC-network and L-network twice in a fine-tuned manner. This is an online method that randomly draws training data from new test environments while learning exploration policy. Generalized training hurts the accuracy of these networks, but this reduction is acceptable for visual perception and navigation tasks (staying above 90%), and the training process can be performed in a 2.5 M interaction (equivalent to a pretraining phase) and does not interfere with the agent’s exploration of the mazes.

**Table 4:** The secondary training results for the TC-network and L-network.

Method	Environment	TC-network (%)	L-network (%)
Pre-training	Parameter selection	92.36%	93.78%
	Maze-1	84.52%	89.84%
	Maze-2	85.14%	87.43%
	Maze-3	78.32%	89.15%
Targeted training	Maze-1	93.16%	93.12%
	Maze-2	92.67%	94.58%
	Maze-3	92.03%	93.24%
Generalization training	Maze-1/Maze-2	90.89%	92.43%
	Maze-1/Maze-3	91.35%	92.52%
	Maze-2/Maze-3	90.62%	91.97%
	Maze-1/Maze-2/Maze-3	90.28%	91.64%

#### Learning exploration with fine-tuning method

In the previous section, we showed that the ICM module and our approach can guide agents to explore the environment efficiently. However, this policy is learned from beginning to end in a maze, and we wonder if the fine-tuning method that plays an important role in the generalization training of TC-network and L-network is useful for learning exploration policy. To investigate this issue, we take as input the exploration policy obtained in the parameter selection experiment of the reward function, fine-tune them with intrinsic motivation and extrinsic reward, and compare their training effects with those of the basic method (results produced by learning from scratch).

##### Fine-tuning with intrinsic motivation

**Figure 17:**
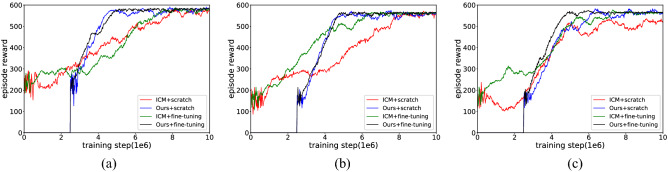
The experimental results of fine-tuning with intrinsic motivation. (**a**) Maze-1 experiment results. (**b**) Maze-2 experiment results. (**c**) Maze-3 experiment results.

Figure 17 (results are averaged over the top 5 random hyperparameters) shows that both ICM and our method can end random exploration earlier and obtain a more stable control policy after training in combination with the fine-tuning method, but there are differences in the effects of the fine-tuning method on exploration policy across the test mazes. From Table 5 (results are averaged over the 5 best performances in the learning process) we see that in Maze-1 there is no significant difference between the scratch learning mode and the fine-tuned learning mode, both in terms of the rewards achieved and the number of interactions required to cover the environment, but we also find something interesting in the learning curve. Due to the relatively simple structure of Maze-1, the exploration policy can be quickly acquired from scratch. The fine-tuning approach, in which behaviour such as wall walking and obstacle avoidance hidden in its initial parameters, instead results in some mismatches in the early training phase. In Maze-2, the fine-tuning approach significantly speeds up the training efficiency of ICM and allows the policy to converge with fewer interactions compared to learning from scratch, but its contribution to our approach is weak. The role of fine-tuning can be better illustrated in Maze-3, and this impact can be seen in two main ways: one is the performance of the ICM module, where the exploration efficiency increases again after the first policy stabilization; the other is the application of fine-tuning to further reduce the number of interactions needed to encode the maze.

**Table 5:** The experimental results of fine-tuning with intrinsic motivation.

Environment	Method	Reward	The maximum exploration ratio within an episode (%)	The interaction required to encode environment (training step/1–6)
Maze-1	ICM + scratch	584.59	100.00	7.93
	Ours + scratch	586.32	100.00	4.72
	ICM + fine-tuning	585.16	100.00	7.58
	Ours + fine-tuning	585.45	100.00	5.14
Maze-2	ICM + scratch	567.28	100.00	8.07
	Ours + scratch	571.87	100.00	5.15
	ICM + fine-tuning	566.34	100.00	6.49
	Ours + fine-tuning	568.25	100.00	4.81
Maze-3	ICM + scratch	532.27	91.64	$\infty$
	Ours + scratch	579.65	100.00	6.54
	ICM + fine-tuning	573.49	100.00	7.23
	Ours + fine-tuning	572.86	100.00	4.73

Most importantly, the experimental results show that the fine-tuning approach cannot always play a positive role and sometimes interferes with the learning process, especially in simple environments. In contrast, the fine-tuning approach works more prominently in complex environments, where it can use pretrained policy as input to guide the agent to better adapt to the new environment.

##### Fine-tuning with extrinsic rewards

The reader should keep in mind that the present experiment placed extrinsic rewards in the test setting and used them as drivers to guide exploration. Extrinsic rewards were in the form of goals (Fig. 12b, value + 10) and apples (Fig. 12c, value + 1), whose positions were fixed within an episode and varied randomly across episodes. If the goal is reached, the agent respawns to a new starting position and must explore the maze again, with the performance of each method still measured by the uniform reward it receives within an episode (the area explored was calculated by the count-based method).

**Figure 18:**
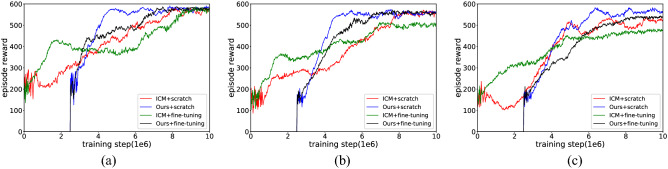
The experimental results of fine-tuning with extrinsic reward. (**a**) Maze-1 experiment results. (**b**) Maze-2 experiment results. (**c**) Maze-3 experiment results.

We note some particular results in Fig. 18 (results are averaged over the top 5 random hyperparameters) and Table 6 (results are averaged over the 5 best performances in the learning process). Compared to the former method, using extrinsic rewards to conduct fine-tuning reflects a more negative effect; it not only slows down the training process but also confuses the difference between exploration and navigation. This performance impairment is at the root of the policy change because touching the goal can be perceived as achieving a large intrinsic reward during exploration, which makes the state containing the extrinsic attractive reward and the agent wants to reach it consistently. Additionally, since the agent is reset to a new starting position when reaching the goal, the purpose of fine-tuning appears to be to find the goal rather than to explore the environment. This is why agents can quickly gain great rewards in the early training stage, but still need more interaction to complete the exploration, a phenomenon that is very prominent in Maze-1 and Maze-2. The worst happens in Maze-3, where there is no way to cover the entire environment in one episode.

**Table 6:** The experimental results of fine-tuning with extrinsic reward.

Environment	Method	Reward	The maximum exploration ratio within an episode (%)	The interaction required to encode environment (training step/1–6)
Maze-1	ICM + scratch	584.59	100.00	7.93
	Ours + scratch	586.32	100.00	4.72
	ICM + fine-tuning	583.74	100.00	9.13
	Ours + fine-tuning	586.56	100.00	7.24
Maze-2	ICM + scratch	567.28	100.00	8.07
	Ours + scratch	571.87	100.00	5.15
	ICM + fine-tuning	514.63	89.46	$\infty$
	Ours + fine-tuning	569.44	100.00	6.83
Maze-3	ICM + scratch	532.27	91.64	$\infty$
	Ours + scratch	579.65	100.00	6.54
	ICM + fine-tuning	483.16	82.95	$\infty$
	Ours + fine-tuning	542.68	92.63	$\infty$

Thus, extrinsic rewards, which are specific objects in the environment, tend to be limited in number and are better suited to lead agents to goal-driven behaviour. In contrast, intrinsic motivation is distributed throughout the state space, and this drive can propel the agent to reach unfamiliar states in the environment and obtain a goal-independent exploration policy.

#### “Noisy-TV” experiment

In the above text, we observe that the ICM method outperforms other baselines and achieves almost the same performance as our method in the first two test mazes. However, the “couch potato” problem, shown in the noisy-TV experiment, remains a hard problem for this prediction-based curiosity approach. Our method relies on agent observation and memory to guide exploration, and this experiment aims to provide more evidence to verify whether it is more robust to stochastic objects.

The noisy-TV experiment was implemented as follows. In all test environments, the TV was on the agent’s frontal display, and its position was fixed within an episode and randomly reset in different episodes. At each step, a random image with a resolution of $21 \times 21$ is displayed on the TV screen, which is independent of the agent’s actions and occupies one of the four quadrants observed by the agent, and each pixel in the image is sampled from $\left[ {0,255} \right]$ uniformly sampled.

**Figure 19:**
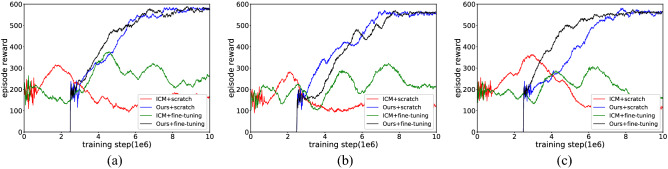
The experimental results of “Noisy-TV”. (**a**) Maze-1 experiment results. (**b**) Maze-2 experiment results. (**c**) Maze-3 experiment results.

The experimental results collected in Fig. 19 (results are averaged over the top 5 random hyperparameters) and Table 7 (results are averaged over the 5 best performances in the learning process) show that the performance of both the ICM and our method decreases after adding the randomness source, and it is the ICM that is most affected. While learning from scratch, ICM quickly exhausts its curiosity and stops exploring, and the fine-tuning approach can facilitate exploration to some extent, but the resulting policy is still unsatisfactory. You can see that some parts of the state space cannot be modelled at all, such as leaves blowing in a breeze or the noisy-TV used in this experiment. Their prediction errors remain high and show an irresistible attraction to the agent, which causes the ICM approach to fall into the curiosity trap and degenerate into undesired behaviour. It is clear that the motion of the leaves and the images of the TV are insignificant for exploration, and it is useless for the agent to continue to be curious about them. Therefore, we searched for curiosity based on memory rather than prediction. Relying on comparisons with the past, the agent does not remain curious about such random objects and overcomes the “couch potato” problem. The experimental results show that our method allows reasonable exploration of the environment and acquisition of complete memory for all tested mazes, although the presence of noisy-TV slows down learning. In addition, other causes of environmental randomness will be further discussed in our future work.

**Table 7:** The experimental results of “Noisy-TV”.

Environment	Method	Reward	The maximum exploration ratio within an episode (%)	The interaction required to encode environment (training step/1–6)
Maze-1	ICM + scratch	315.62	53.86	$\infty$
	Ours + scratch	582.74	100	7.58
	ICM + fine-tuning	374.52	64.05	$\infty$
	Ours + fine-tuning	586.43	100	8.67
Maze-2	ICM + scratch	279.68	48.71	$\infty$
	Ours + scratch	565.32	100	6.93
	ICM + fine-tuning	317.54	56.18	$\infty$
	Ours + fine-tuning	566.73	100	7.75
Maze-3	ICM + scratch	362.49	63.28	$\infty$
	Ours + scratch	577.86	100	7.69
	ICM + fine-tuning	305.47	54.72	$\infty$
	Ours + fine-tuning	572.63	100	8.12

### Goal reaching experiment

In navigational tasks, we compare our approach with a set of navigation models equipped with the DRL framework, including DQN, DRQN, Nav A3C, and an enhanced version of Nav A3C + D_2_L. Since space topological cognition is done during exploration, it can be used directly to achieve the goal. For a fair comparison, we trained these models using the same training steps used to learn the exploration policy and save the trained models as baselines. The environment applied in section “Fine-tuning with extrinsic rewards” was used to train these models and conduct subsequent goal reaching experiments. Instead of using the extrinsic reward obtained by the agent within an episode (5000 time steps) to evaluate navigation efficiency, we used the percentage of success in reaching the goal at different time steps as a benchmark for comparison.

#### Static maze experiment

In this experiment, the structure in the environment does not change except for the positions of the agent and the target, and we ensure that all operations in our approach are valid. Target localization is performed only once at the beginning of an episode, and the only remaining computational operation is the agent’s self-localization.

**Figure 20:**
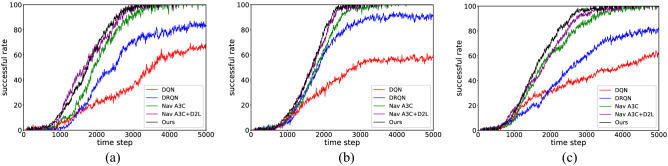
The goal reaching experimental results in static mazes. (**a**) Maze-1 experiment results. (**b**) Maze-2 experiment results. (**c**) Maze-3 experiment results.

As shown in Fig. 20 (results are averaged over 30 times), due to the randomness of the target location across episodes, DQN simply obtains some reactive or wall-following policy held by the encoder weights, which makes it difficult to derive an effective navigation policy for this method and makes it more desirable to enter the target state when the agent is localized near the target. The DRQN model is equipped with an LSTM and compensates for the memory deficit of the DQN, which remembers the target location and returns as many times as possible in an episode, but it requires a large number of time steps to find the target for the first time. Additional information (agent-relative velocity, action and reward) is used in the Nav A3C model, and these inputs further improve the navigation efficiency. When combined with ground-truth depth map and loop closure, it achieves a similar performance to our approach in the first two test mazes. However, this phenomenon changes in Maze-3 because the structure of this environment is more complex, the contribution of depth information to the selection of actions decreases, and the navigation efficiency of Nav A3C + D_2_L shows a decreasing trend, while our method still maintains an efficient navigation policy.

Table 8 (the results are averaged over random 30 episodes in the testing process) presents the navigation performance of each model in more detail, and we note some particular results for different test environments. First, there is no doubt that the memory function is crucial for navigation, and the absence of a memory function significantly reduces the number of times an agent reaches a goal and the rewards it achieves within an episode. Such a result is more pronounced in environments requiring explicit memory, such as Maze-2, where the DRQN model has almost twice as much reward as the DQN model. Second, although the DRQN model outperforms the DQN model in terms of navigation efficiency in all test mazes, the DRQN model still requires a significant number of time steps to reach the goal. We believe the reason for this is due to the inability of the agent to clearly identify some observations, which supports the addition of additional inputs and depth information to consolidate the mapping relationship between states and actions. Finally, it can be seen from the collected data that the map-less approach is an alternative to the map-based approach in simple environments, but the performance of the former decreases in Maze-3, indicating that the map-based approach can better handle the increased complexity of the state space.

**Table 8:** The goal reaching experimental results in static mazes.

Environment	Method	The number of times to reach the goal within an episode	The reward achieved within an episode
Maze-1	DQN	0.65	8.74
	DRQN	0.82	11.53
	Nav A3C	1.39	17.28
	Nav A3C + D_2_L	1.72	20.34
	Ours	1.68	19.87
Maze-2	DQN	0.53	7.82
	DRQN	0.94	13.16
	Nav A3C	1.57	18.45
	Nav A3C + D_2_L	2.28	24.93
	Ours	2.15	23.75
Maze-3	DQN	0.62	8.53
	DRQN	0.78	10.65
	Nav A3C	1.18	14.29
	Nav A3C + D_2_L	1.32	17.84
	Ours	1.67	19.58

#### Dynamic blockage experiment

**Figure 21:**
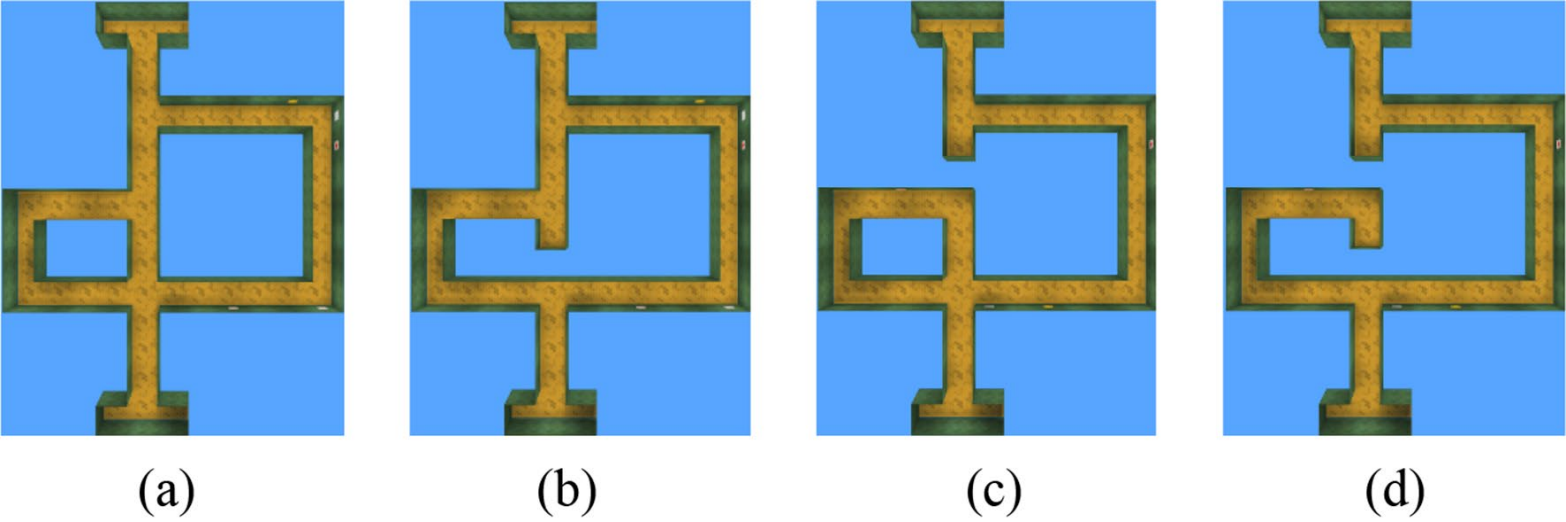
Dynamic blockages experimental environment. (**a**) Non-blocking. (**b**) Block at point A. (**c**) Block at point B. (**d**) Block at point A and B.

In this section, we discuss the performance of each method in state space with dynamic blockages. The test environments have the same layout as Maze-1 but with additional blockages in the maze, whose locations are shown in Fig. 21 (there are still available paths within the maze). We then use the space topological cognition and navigation model obtained in Maze-1 to conduct experiments in the environments shown in Fig. 21.

**Figure 22:**
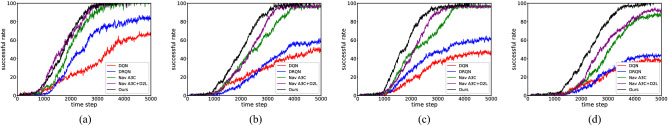
The goal reaching experimental results in dynamic blockages environments. (**a**) Non-blocking experiment results. (**b**) Block at point A experiment results. (**c**) Block at point B experiment results. (**d**) Block at point A and B experiment results.

In fact, as seen in Fig. 22 (the results are averaged over 30 times), adding dynamic blockages to Maze-1 affects the navigation efficiency of all methods. However, the impact of blockages is fatal for map-less methods compared to map-based methods. For DQN and DRQN, these methods prefer to use reactive behaviour to find the target. Therefore, if the agent is separated from the target by a blockage, it will stall at the blockage until the end of the episode, which makes the success rate much lower. Nav A3C and Nav A3C + D_2_L are also affected, and both of them lose the guarantee of reaching the target when the blockage appears at position A or B, although they have excellent performance in the non-blocking state space. This degradation in performance is more obvious in the last environment because there are two blockages, and the probability of the agent and the target being separated by the blockage increases significantly. Similarly, the success rate of our approach decreases because our approach requires more time steps to guide the agent around the blockage.

Table 9 (results are averaged over 30 random episodes during the test) shows the same trend as in Fig. 22. For all methods the number of times they reach the goal and the rewards achieve decreases as blockages appear, with the methods without maps being the most affected. In particular, the rewards of DQN and DRQN decrease by nearly 30% when blockages are placed at either location A or B and by nearly half in environments that included two blockages. Additionally, the presence of blockages exposes the shortcomings of Nav A3C and Nav A3C + D_2_L, especially in the case where the agent and the target are placed at both ends of the blockage, where agents constantly attempt to break through the blockage, resulting in a significant decrease in their frequency of reaching the target. Relying on the memory of the environment structure and dynamic path planning mechanism, our approach ensures that the agent can find an available path to reach the goal. Specifically, the agent can correct the topological memory during navigation and use it to bypass blockages A and B. However, due to the increased navigation distance, our approach often requires the entire episode to reach the goal, which results in lower rewards.

**Table 9:** The goal reaching experimental results in dynamic blockages environments.

Environment	Method	The number of times to reach the goal within an episode	The reward achieved within an episode
Non-blocking	DQN	0.65	8.74
	DRQN	0.82	11.53
	Nav A3C	1.39	17.28
	Nav A3C + D_2_L	1.72	20.34
	Ours	1.68	19.87
Block at A	DQN	0.51	7.34
	DRQN	0.58	8.06
	Nav A3C	0.95	11.52
	Nav A3C + D_2_L	0.93	11.48
	Ours	1.28	14.73
Block at B	DQN	0.47	7.15
	DRQN	0.63	8.32
	Nav A3C	0.96	11.86
	Nav A3C + D_2_L	0.95	11.54
	Ours	1.32	15.06
Block at A and B	DQN	0.38	5.74
	DRQN	0.43	6.28
	Nav A3C	0.85	10.64
	Nav A3C + D_2_L	0.89	11.37
	Ours	1.15	13.21

## Conclusion

In this work, we proposed a novel navigation architecture consisting of intrinsic motivation exploration and space topological cognition. The goal of the first component of our approach is to explore the environment, while the goal of the other component is to encode the environment, and they are specifically designed to work together. Experimental results and analyses highlight the role of reward function and training patterns in learning exploration policy. Additionally, we investigated the navigation performance of agents equipped with space topological cognition in static and semi-dynamic environments.

Our approach is inspired by the cognitive mechanism of animals, which can explore their environment and encode its structure in a synchronized manner. For AI agents, to accomplish spontaneous exploration from raw visual inputs, we used DRL as the basic learning framework and allowed agents to create rewards for themselves. Considering the limitations of prediction-based exploration methods, our reward function is computed based on episode memory and includes two types of novel rewards. Space topological cognition is populated by waypoints during exploration, which is also found in episode memory. Such spatial cognition can be used to gradually cover the environment by integrating exploration sequences and as a planning module for the navigation system.

Although our approach successfully learns exploration policy through the end-to-end DRL framework, the capacity of the 1-layer LSTM can be stretched in very large environments due to its limited memory. In the future, it will be important to increase the LSTM or utilize external memory to improve the capability of our learned model. Moreover, in our approach, the size of spatial cognition grows linearly with the area explored. Again, this can become a problem when navigating in very large environments. A possible solution is secondary sampling, where only the most informative or discriminative waypoints are stored. Finally, we see future work in migrating our approach to the real world and comparing it with vision-based SLAM methods.

## Data Availability

The datasets used or analyzed during the current study are available from the corresponding author on reasonable request.

## References

[1] Oudeyer, P.Y. Computational theories of curiosity-driven learning. arXiv:1802.10546 (2018).

[2] Tolman EC (1948). Cognitive maps in rats and men. Psychol. Rev..

[3] Mirowski, P., Pascanu, R., Viola, F., Soyer, H., Ballard, A.J., Deil, M., Goroshin, R., Sifre,L., Kavukcuoglu, K., Kumaran, D., & Hadsell, R. Learning to navigate in complex environments. arXiv:1611.03673 (2017).

[4] LeCun Y, Bengio Y, Hinton G (2015). Deep learning. Nature.

[5] Oh, J., Chockalingam, V., Singh, S. P., & Lee, H. Control of memory, active perception, and action in Minecraft. arXiv:1605.09128 (2016).

[6] Zhu, Y., Mottaghi, R., Kolve, E., Lim, J. J., Gupta, A., Fei-Fei, L., & Farhadi, A. Target-driven visual navigation in indoor scenes using deep reinforcement learning, in *2017 IEEE International Conference on Robotics and Automation (ICRA)* 3357–3364 (2016).

[7] Mnih, V., Badia, A.P., Mirza, M., Graves, A., Lillicrap, T.P., Harley, T., Sliver, D., & Kavukcuoglu, K. Asynchronous methods for deep reinforcement learning. arXiv:1602.01783 (2016).

[8] Gers FA, Schmidhuber J, Cummins F (2000). Learning to forget: Continual prediction with LSTM. Neural Comput..

[9] Jaderberg, M., Mnih, V., Czarnecki, W. M., Schaul, T., Leibo, J. Z., Sliver, D., & Kavukcuoglu, K. Reinforcement learning with unsupervised auxiliary tasks. arXiv:1611.05397 (2016).

[10] Ye, X., Lin, Z., Li. H., Zheng, S., & Yang, Y. Active object perceiver: Recognition-guided policy learning for object searching on mobile robots. arXiv:1807.11174v1 (2018).

[11] Yang, W., Wang, X., Farhadi, A., Gupta, A., & Mottaghi, R. Visual semantic navigation using scene priors. arXiv:1810.06543 (2018).

[12] Devo A, Mezzetti G, Costante G, Fravolini ML, Valigi P (2020). Towards generalization in target-driven visual navigation by using deep reinforcement learning. IEEE Trans. Robot..

[13] Berlyne, D. E. *Conflict, Arousal and Curiosity* 38–54 (McGraw-Hill Book Company, 1960).

[14] Harlow FH (1950). Learning and satiation of response in intrinsically motivated complex puzzle performances by monkeys. J. Comp. Physiol. Psychol..

[15] Sylva, K., Bruner, J. S., & Jolly, A. *Play: Its role in development and evolution* 279–292 (Penguin Books Ltd, 2017).

[16] Bellemare, M. G., Srinivasan, S., Ostrovski, G., Schaul, T., Saxton, D., & Munos, R. Unifying count-based exploration and intrinsic motivation, in *NIPS* (2016).

[17] Ostrovski, G., Bellemare, M.G., Oord, A. V. D., & Munos, R. Count-based exploration with neural density models. arXiv:1703.01310 (2017).

[18] Tang, H., Houthooft, R., Foote, D., Stooke, A., Chen, X., Duan, Y., Schulman, J., Turck, F. D., & Abbeel, P. Exploration: A study of count-based exploration for deep reinforcement learning, in *NIPS* (2017).

[19] Houthooft, R., Chen, X., Duan, Y., Schulman, J., Turck, F. D., & Abbeel, P. Vime: Variational information maximizing exploration, in *NIPS* (2016).

[20] Fu, J., Co-Reyes, J. D., & Levine, S.: EX2: Exploration with exemplar models for deep reinforcement learning, in *NIPS* (2017).

[21] Pathak, D., Agrawal, P., Efros, A. A., & Darrell, T. Curiosity-driven exploration by self-supervised prediction. arXiv:1705.05363 (2017).

[22] Pritzel, A., Uria, B., Srinivasan, S., Puigdomenech, A., Vinyals, O., Hassabis, D., Wierstra, D., & Blundell, C. Neural episode control. arXiv:1703.01988 (2017).

[23] Sermanet, P., Lynch, C., Chebotar, Y., Hsu, J., Jang, E., Schaal, S., & Levine, S. Time-contrastive network: Self-supervised learning from video. arXiv:1704.06888 (2018).

[24] Aytar, Y., Pfaff, T., Budden, D., Paine, T. L., & Wang, Z. Playing hard exploration games by watching youtube. arXiv:1805.11592 (2018).

[25] Cadena C, Carlone L, Carrillo H, Latif Y, Scaramuzza D, Reid I, Leonard JJ (2016). Past, present, and future of simultaneous localization and mapping: Toward the robust-perception age. IEEE Trans. Robot..

[26] Bhatti, S., Desmaison, A., Miksikm, O., Nardelli, N., Siddharth, N., & Torr, P. H. S. Playing doom with SLAM-augmented deep reinforcement learning. arXiv:1612.00380 (2016).

[27] Parisotto, E., & Salakhutdinov, R. Neural map: Structured memory for deep reinforcement learning. arXiv:1702.08360 (2017).

[28] Gupta, S., Tolani, V., Davidson, J., Levine, S., Sukthankar, R., & Malik, J. Cognitive mapping and planning for visual navigation. arXiv:1702.3920 (2019).

[29] Mnih V, Kavukcuoglu K, Sliver D, Rusu AA, Veness J, Bellemare MG, Graves A, Riedmiller MA, Fidjeland A, Ostrovski G, Petersen S, Beattie C, Sadik A, Antonoglou I, King H, Kumaran D, Wierstra D, Legg S, Hassabis D (2015). Human-level control through deep reinforcement learning. Nature.

[30] Williams RJ (1992). Simple statistical gradient-following algorithms for connectionist reinforcement learning. Mach. Learn..

[31] Nachum, O., Norouzi, M., Xu, K., & Schuurmans, D. Bridging the gap between value and policy based reinforcement learning. arXiv:1702.08892 (2017).

[32] Sutton, R. S., & Barto, A. G. *Reinforcement learning: An introduction* 215–260 (The MIT Press, 1998).

[33] He, K., Zhang, X., Ren, S., & Sun, J. Deep residual learning for image recognition. arXiv:1512.03385 (2015).

[34] Friston K, Fitzgerald T, Rigoli F, Schwartenbeck P, Pezzulo G (2017). Active inference: A process theory. Neural Comput..

[35] Forestier, S., & Oudeyer, P. Y. Modular active curiosity-driven discovery of tool use, in *2016 IEEE/RSJ International Conference on Intelligent Robots and Systems (IROS)* 3965–3972 (2016).

[36] Salge C, Glackin C, Polani D (2014). Changing the environment based on empowerment as intrinsic motivation. Entropy.

[37] Little DY, Sommer FT (2013). Learning and exploration in action–perception loops. Front. Neural Circuits.

[38] Sutton, R. S. Integrated architectures for learning, planning, and reacting based on approximating dynamic programming, in *Proceedings of the Seventh International Conference on Machine Learning* 226–224 (1995).

[39] Sigaud, O., & Stulp, F. Policy search in continuous action domains: An overview. arXiv:1803.04706 (2018). 10.1016/j.neunet.2019.01.011 30780043

[40] Moser EI, Kropff E, Moser MB (2008). Place cells, grid cells, and the brain’s spatial representation system. Annu. Rev. Neurosci..

[41] Kirichuk VS, Kosykh VP, Popov SA, Shchikov VS (2014). Suppression of a quasi-stationary background in a sequence of images by means of interframe processing. Optoelectron. Instrument. Data Process..

[42] LeCun Y, Bengio Y, Hinton G (2015). Deep learning. Nature.

[43] Cormen, T. H., Leiserson C. E., Rivest, R. L., & Stein, C. *Introduction to Algorithms,* 3rd ed, 658–664, 682 (The MIT Press, 2005).

[44] Beattie, C., Leibo, J.Z., Teplyashin, D., Ward, T., Wainwright, M., Kuttler, H., Lefrancq, A., Green, S., Valdes, V., Sadik, A., Schrittwieser, J., Anderson, K., York, S., Cant, M., Cain, A., Bolton, A., Caffney, S., King, H., Hassabis, D., Legg, S., & Petersen, S. Deepmind lab. arXiv:1612.03801 (2016).

[45] Schulman, J., Levine, S., Moritz, P., Jordan, M. I., & Abbeel, P. Trust region policy optimization. arXiv:1502.05477 (2017).

[46] Hausknecht, M., & Stone, P. Deep recurrent Q-learning for partially observable MDPs. arXiv:1507.06527 (2017).

[47] Kingma, D. P., & Ba, J. Adam: A method for stochastic optimization. arXiv:1412.6980 (2017).

